# Perceived feasibility of sward management options in permanent grassland of Alpine regions and expected effects on delivery of ecosystem services

**DOI:** 10.1007/s10668-022-02899-y

**Published:** 2023-01-05

**Authors:** Gabriele Mack, Nadja El Benni, Martina Spörri, Olivier Huguenin-Elie, Sophie Tindale, Erik Hunter, Paul Newell Price, Lynn J. Frewer

**Affiliations:** 1 Sustainability Assessment and Agricultural Management, Agroscope, Tänikon, Ettenhausen, Switzerland; 2 Animal Production Systems and Animal Health, Agroscope, Zurich, Switzerland; 3 Centre for Rural Economy, School of Natural and Environmental Sciences, Newcastle University, Newcastle Upon Tyne, UK; 4 Department of People and Society, Swedish University of Agricultural Sciences, Uppsala, Sweden; 5 ADAS, Mansfield NG20 9PD, UK

**Keywords:** Delphi, Farmer interviews, Mountain agriculture, Sward renewal, Overseeding, Rising plate meters

## Abstract

Agriculture in Alpine regions plays an important role for multiple ecosystem services (ES) supplied from permanent grassland (PG). This paper investigates the feasibility of sward renewal, overseeding, and rising plate meters on PG for the Swiss Alpine region and analyses their expected effects on ES supply. Sward renewal and overseeding are management options implemented in response to a decline of grassland yields and nutritive value or sward damage. Rising plate meters focus on increased grass utilisation for improving profitability of grassland farms in a sustainable manner. The aim was to improve the understanding which of these three PG management practices could be promoted to deliver a wide range of agricultural and non-agricultural ESs in the Swiss Alpine region. Through interviews with 75 farmers (including organic and intensive/extensive non-organic farmers) and a Delphi-methodology on a panel of experts (*N* = 10 experts with different expertise), we found that sward renewal is perceived to have negative effects on biodiversity, carbon storage, flood control, prevention of soil erosion, and prevention of loss of organic matter in Alpine regions. Therefore, sward renewal should not be promoted, although about half of the farmers interviewed had already carried out the practice on plots following severe sward damage in Alpine regions. Overseeding is perceived to have positive effects on biodiversity, prevention of soil erosion, and grass production. Thus, the high level of overseeding that is currently practiced in the Swiss Alpine region is probably sustainable. Rising plate meters do not play a significant role in PG management in the Alpine region because calibration in PG with diverse grassland botanical composition in the Alpine region is too difficult.

## Introduction

In Europe, permanent grassland (PG) accounts for about one-third of the total utilised agricultural area (Estel et al., 2018). PG is key to the supply of multiple important ecosystem services (ES) (Schils et al., 2020). If PG is properly managed, it provides not only agricultural but also non-agricultural services, such as water supply and flow regulation, carbon storage, erosion control, climate mitigation, pollination and cultural ES (Bengtsson et al., 2019; Marzetti et al., 2011). PG maintenance and the supply of its ES are under threat globally due to land-use changes, climate change, agronomic intensification and land abandonment (Griffin-Nolan et al., 2019; Hecht et al., 2016; Schils et al., 2022; Taube et al., 2014). PG in Alpine regions is sensitive and vulnerable to these influences (Berauer et al., 2019; Steininger & Weck-Hannemann, 2002; Wen et al., 2013; Wu et al., 2017).

Agriculture in the Alpine Arc plays a vital role in maintaining the broad range of ES from PG (MacDonald et al., 2000; Nadal-Romero et al., 2021; Santini et al., 2013), while at the same time facing issues such as low productivity and high production costs due to topographic, soil and climatic constraints within the Alpine region (Mann, 2013; Huber et al., 2015). In this context, PG management practices such as (1) sward renewal that is, in some regions, applied in response to a decline in grassland yield and nutritive value or sward damage (Buchen et al., 2017; Creighton et al., 2011; Klaus et al., 2018), (2) overseeding in order to increase forage production and quality (Bondaruk et al., 2020; Jaurena et al., 2016), and (3) rising plate meters to monitor and predict grass growth to increase grass utilisation by grazing livestock (Beukes et al., 2019; French et al., 2015) might be adopted in vulnerable mountain regions to improve efficiencies. However, there is a lack of knowledge how the uptake of these three PG management practices influence the wide range of ES from PG in the Alpine region. Furthermore, knowledge about the extent to which PG farmers in the Alpine regions adopt these management practices and their reasons for doing so is lacking. Greater knowledge on the adoption of sward renewal, overseeding, and rising plate meters in Alpine regions and their impacts on different ES improve the understanding which of the three PG management practices should be supported or prevented in the Swiss Alpine region.

The purpose of this research is to explore the feasibility of different PG management practices (i.e. sward renewal, overseeding and rising plate meter use) and their expected impacts on ES (e.g. provisioning, regulating, supporting and cultural ES) in Alpine regions. Based on a case study in the Swiss Alpine region, interviews with 75 farmers provide information about the reasons for adopting or rejecting these specific PG management practices in mountain agriculture. Delphi methodology with experts was applied to assess the feasibility and expected effect of these management practices on different ES for PG in the Swiss Alpine region, in order to provide recommendations regarding which of the three PG management practices could be promoted to deliver a wide range of ESs in the Swiss Alpine region.

Previous research on ES delivery from mountain grassland regions has primarily focused on threats due to climate and land-use change (Griffin-Nolan et al., 2019; Schirpke et al., 2017; Taube et al., 2014, Runting et al., 2017; Tasser et al., 2017; Hanaček et al., 2018), or investigated how socio-economic conditions and agricultural policy affect ES delivery in mountain regions (Briner et al., 2013; Huber et al., 2017; Jaligot et al, 2019). To the best of our knowledge, there are no previous studies on PG management strategies such as sward renewal, overseeding, and rising plate meters and how they influence ES supply from PG in Alpine regions.

The contribution of our paper is twofold: First, it contributes to the growing body of research on the effects of changing human interventions on ES delivery in Alpine regions. Second, the paper contributes to the growing body of published literature focusing on the uptake of sward manipulating practices as well as measures to monitor and predict grass growth.

## Description of the case study region

**Fig. 1:**
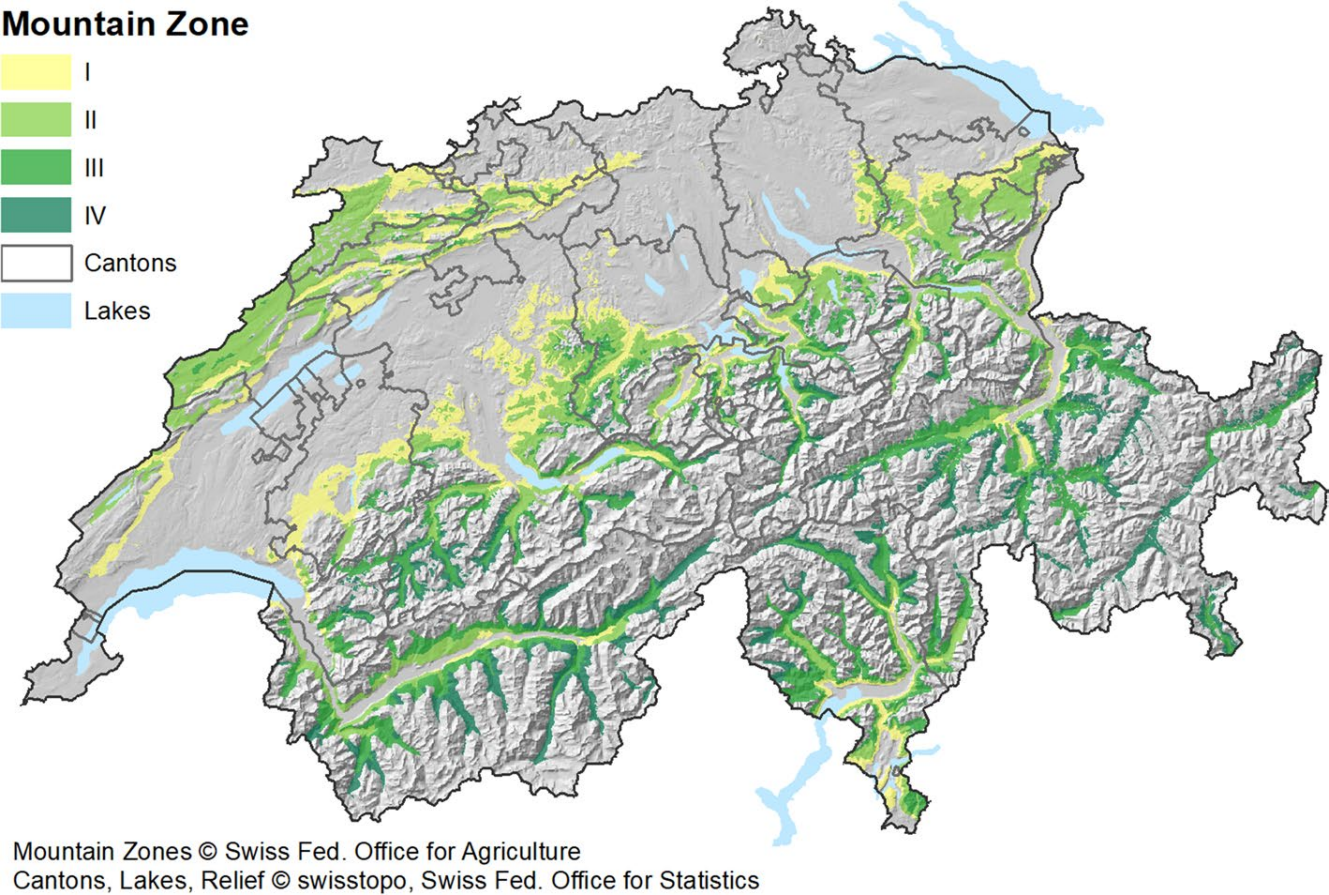
Relief map of the case study region: Swiss mountain zones I–IV

The case study region represents the utilised agricultural area (UAA) in the Swiss mountain zones I–IV. Figure 1 provides a relief map of the Swiss mountain zones. With increasing zone number, natural conditions for agricultural production are becoming more difficult due to increasing altitude and land slope (FOAG, 2020). Grasslands within the UAA reach altitudes of over 1600 m a.s.l. The case study region excludes Alpine summer pastures, which are governed by other regulations (Mack et al., 2013).

In Swiss mountain zones, PG covers 88% of the UAA (FOAG, 2018). The environmental conditions of the Swiss mountain region together with Swiss agricultural policy are the main drivers for this dominance of grasslands. Many areas of the case study region have favourable conditions for grass growth due to a relatively even rainfall distribution throughout the year. Fertilised permanent grasslands can produce as much as 14 tonnes of dry matter per hectare annually (Huguenin-Elie et al., 2017). Grassland yields markedly decrease with increasing altitude due to the shortening of the growing season, but if mountain grassland is not too intensively used, it makes an important contribution to biodiversity conservation (Huguenin-Elie et al., 2018). The topography of the Swiss mountain region results in a large range of climatic conditions, which, combined with a wide range of soil types, results in very diverse environmental conditions. For instance, annual precipitation ranges from 850 to 2160 mm, without considering the extreme situations (and this covers 95% of the permanent grasslands; Lüscher et al., 2019). On top of this environmental variability comes large differences in management intensity at farm scale (among the grassland fields within a farm). Part of the grassland area produces high-quality forage to satisfy demand for grass based ruminant production (Ineichen et al., 2016); however, this also requires intensive management such as the harvesting of forage at early development states with high nutrient content and the need for appropriate fertilisation. In contrast, agri-environmental schemes promote maintenance of extensive grasslands within each farm (Mack et al., 2020). Thus, in the Swiss mountain region, each farmer manages a variety of grassland types, with different defoliation frequencies and fertilisation regimes. Reference values of yield levels for different grassland types with increasing altitude in Switzerland are provided in appendix (Table 3).

## Description of PG management practices

*Complete sward renewal* involves cultivating or using herbicide on the existing sward to eliminate the existing plant species, and the introduction of a selection of desired plant species by broadcast sowing or drilling. It is used when the existing sward is not meeting current land management objectives, such as when the current sward has less than 50–60% of the desired or sown species (AHDB, 2019). These could be productive species or species that support pollination, biodiversity or deeper rooting for drought resilience. Sward renewal is generally used to increase productivity and/or biodiversity. In the first years following sowing, this should increase the proportion of sown species, which may be grasses, herbs and/or legumes and, where increasing biodiversity is the main objective of the land management activities, regionally native species. However, experience has shown that sward renewal does not usually achieve stable plant communities and that the sown species sharply decline following the first three to five years after sowing (Brophy et al., 2017). In Switzerland, destroying permanent grassland using herbicides requires special authorisation from the competent authorities.

*Overseeding or oversowing* involves broadcasting or slot seeding seeds of desired species within an existing plant community. It is used when a land manager wants to retain the species in the existing sward but would like to increase the proportion of certain desired species or introduce other species. It is recommended when the proportion of desired species in the current sward is above 15% but less than 50–60% (AHDB, 2019; Natural England, 2010a, 2010b). These desired species could be productive species or species that support pollination, biodiversity or deeper rooting for drought resilience. The aim may be to increase productivity or botanical diversity (mainly the wildflower component) of species-poor grassland. Cultivating the soil to at least 50% bare soil creates establishment niches for sown species, while retaining some plant cover, but increases the workload and costs of the practice.

*Rising plate meters* are used to measure grassland sward height as a proxy for grass biomass. They measure compressed sward height with a movable plate fastened onto a stick. Measurement values are recorded either manually, mechanically or digitally, and a large number of measurements can be taken in a short time. The user is recommended to follow a ‘W’ path over a field, avoiding atypical areas such as gateways, feeding areas and shaded areas, to measure compressed sward height in about forty equally spaced positions along the ‘W’ (Frame & Laidlaw, 2014). Grass is usually measured every week to assess standing biomass and also just before a grazing cycle. Recent models include GPS modules and wireless technology, which have been used to help with data interpretation and analysis. The relationship between sward height and grass biomass depends on the botanical composition of the grassland. Therefore, proper calibration of the regressions used in the underlying models for estimating biomass is essential. Such calibrations are not currently available for the diversity of grassland types existing in the Alpine region. The information supports decisions on grazing management, including when to start grazing, rotation length, the need for conservation and supplementary feeding, and fertiliser and manure application planning. The current forage supply on pastures (standing biomass) and current growth rates (change in standing biomass from one week to the next) are important parameters for planning grazing management as well as for benchmarking pasture performance.

## Methodological approach

The research presented explores the feasibility of the three management practices including sward renewal, overseeding and rising plate meters. A multi-method approach was applied to analyse the feasibility of these three practices from (1) a farmers’ perspective and (2) experts’ perspectives:

1. We evaluated results from telephone interviews carried out with 75 grassland farmers in 2020. Intensive and extensive farmers (non-organic), as well as organic farmers, were interviewed to analyse the uptake of the three management practices and reasons for adopting or rejecting these practices in mountain agriculture.
2. A Delphi study was carried out to explore the feasibility and the expected effects on ES supply from an experts’ perspective. Ten experts were recruited from four institutions in Switzerland, representing a range of 10 academic disciplines.

### Farmer interviews

Seventy-five farmers from the Swiss Alpine region were interviewed from October to December 2020. Due to the COVID-19 crisis, telephone interviews were conducted. We started the recruitment process of interviewees in February 2020 with a postal mail sent to almost 2000 members of the Swiss Grassland Society (AGFF^1^) in Switzerland. In order to select interviewees, we asked them to fill in a short questionnaire and to indicate whether they were willing to participate in the interviews.

The respondents were grouped into three groups: conventional intensive (stocking rate greater than 1 livestock unit [LU] per ha), conventional extensive (stocking rate smaller than 1 LU per ha) and organic farms. From each group, we selected randomly 25 farms as interview participants (total *N* = 75). Table 4 in Appendix gives an overview of regional, farm and socio-economic characteristics of the three groups of interviewees.

The survey formed part of a broader study assessing the drivers and barriers to adoption of different grassland management practices, as well as tipping points for change in grassland systems. More precisely, in relation to specific PG management practices farmers were asked to rank management options in relation to the likelihood of adoption on their own farm. Relevant management options to this study included: “complete sward renewal with sward destruction (non-selective herbicide spraying or soil ploughing)”, “Overseeding with different grass, herb/legume species or mixtures without complete sward destruction”, “Monitoring grass growth throughout the season using a rising plate meter” on a 7 point Likert scale from “*already in use*; *very likely* [to adopt]; *likely*; *neither likely nor unlikely*; *unlikely*; *very unlikely* and *not possible*”. Additional comments and explanations were recorded.

We analysed the results descriptively based on frequency charts and applied Pearson's Chi-squared (χ^2^) tests to analyse whether significant differences in the uptake of management options were present between intensive, extensive and organic farms.

### Delphi study

Delphi, as classically construed, involves iterated questionnaires being presented anonymously to experts, with controlled feedback between rounds, and the equal weighting of final round responses to produce a group judgement (Linstone & Turoff, 1975). Variations of the method exist, in terms of the number of rounds used, whether or not the first round is structured (quantitative) or unstructured (qualitative), whether the process takes place using paper-and-pencil questionnaires or ‘online’, and whether the process is synchronous or asynchronous (Rowe et al, 1991). Effective application of Delphi in the area of agriculture, and agricultural policy, has been noted (Frewer et al., 2011).

An online Delphi study, using two rounds of questionnaires with anonymised feedback of results between rounds, was conducted with ten experts, who assessed management options, including sward renewal, overseeding and rising plate meters, in terms of the delivery of ES and their feasibility and applicability in the Alpine region of Switzerland. A multidisciplinary expert panel representing a range of 10 academic disciplines was included in the Delphi (Table 1). According to EFSA (2014), the size of such an expert panel should be restricted to the minimum needed to cover the defined expertise profiles. Therefore, ten expert participants for the Delphi study were recruited from four institutions in Switzerland. Experts were selected by the research team for their subject knowledge as well as contextual knowledge of Swiss Alpine regions. The survey took place in September and October 2020.

**Table 1:** Selection of experts and their academic disciplines

Institutions	No of experts	Academic disciplines
Eidgenössische technische Hochschule Zurich, Switzerland (ETH Zurich)	2	Economist, veterinary scientist
Agricultural advisory centre of Switzerland (Agridea)	1	Adviser on digital technologies for ruminant husbandry
Federal research centre for agriculture in Switzerland (Agroscope)	6	Grassland scientist, ecologist, soil scientist, livestock scientist, engineer, animal welfare scientist
Research centre for organic agriculture (Fibl)	1	Social scientist

A modified Delphi technique (Hasson & Keeney, 2011) was used to explore the attitudes of the interdisciplinary group of experts and gather information and opinions in order to obtain the most reliable position of the group (Dalkey & Helmer, 1963). The survey consisted of closed questions, which were answered using Likert scales and open-ended questions (linked to each Likert item), that allowed for elaboration and explanation. The first round of questions focused on the assessment of each management option in relation to its rationale, mechanism of action and outcomes, ES delivery and applicability (Appendix, Table 5). The second round presented anonymised summaries of the results of the first round. This allowed experts the opportunity to clarify or change their opinions based on the answers from the first round.

Of the 10 experts that indicated willingness to participate in the Delphi survey, answers from 9 experts could be used for the analysis. The majority of the experts were very familiar or fairly familiar with the 3 management practices (Appendix, Table 6).

## Results

### Feasibility of sward management options from a farmers’ perspective

**Fig. 2:**
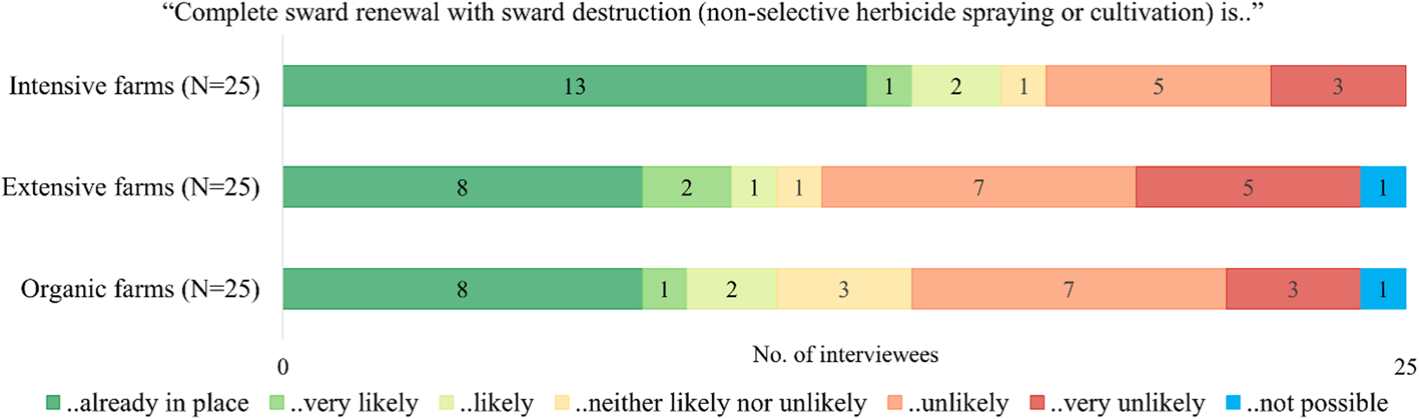
Results of farmer interviews: Uptake of complete sward renewal in the Alpine region of Switzerland (Average stocking rate: Intensive farms = 1.7 LU/ha; Extensive farms = 0.7 LU/ha; Organic farms = 1.1 LU/ha)

Across the 75 farmer interviews, less than half (29) stated that sward renewal was already adopted on PG in Alpine regions. Intensive farms had adopted sward renewal more frequently than organic and extensive farms (Fig. 2). However, the Pearson *χ*2 test did not confirm a significant difference between interviewees with intensive and extensive farms with respect to the current and future uptake of sward renewal. Similarly, there was no significant difference between intensive and organic farms. Results of the Pearson *χ*2 test are reported in Appendix, Table 7.

The reasons for applying sward renewal varied, but were often associated with damage from livestock. For example, one interviewee (intensive farm) mentioned “*I have adopted sward renewal only where the cattle grazing routes pass*” (CH-40). In the extensive farmers’ group, complete sward renewal was associated with more negative connotations, and some farmers expressed the view that sward renewal was only necessary if the grass sward had been severely damaged. Sward renewal was often applied in small areas, e.g. a farmer (extensive farm) stated “*I do not renew a large proportion of the sward, I only fill hollows in order to improve the grassland if the terrain is uneven somewhere* (CH-66)*.* Weather conditions and topography were cited as reasons why farmers are unlikely to take up complete sward renewal. One interviewee stated that “s*ward renewal is not suitable on sites where there is a danger of erosion or a lot of precipitation. I know of farmers who totally lost control of the sward and then renewed it, but that is rather the exception and only on small sites”* (CH-66). Another interviewee (extensive farm) stated “*I once tried to improve a sward by discing it, but that didn't work out due to bad weather conditions. Since then, I have tried as best as possible to avoid gaps in the sward*” (CH-18). Some farmers also mentioned that the topography in Alpine regions may not allow ploughing of PG. One interviewee (intensive farm) stated “*I have taken up sward renewal only on sites where ploughing is possible. I do not know how to destroy the sward when ploughing is not possible, it depends also on the season and the weather*” (CH-31). A number of interviewees (intensive farm) mentioned the limitations of sward renewal compared to overseeding. One interviewee (intensive farm) stated “*I won’t take up sward renewal in the upcoming years because of positive experiences with overseeding*” (CH-38). Another interviewee (intensive farm) stated “*I stopped ploughing up permanent grassland and use other management practices to improve its’ productivity*” (CH-43). Another interviewee (intensive farm) stated “*I had bad experiences with sward renewal and therefore it is very unlikely that I will implement it in future”*. He added “*we did sward renewal once because the grassland was intensively grazed. As a result, the sward suffered from the intensive spreading of manure and grazing. For this reason, we destroyed the sward. But it lasted only for 2–3 years and then the sward was damaged again. Then we started with overseeding and got better grass*” (CH-34).

**Fig. 3:**
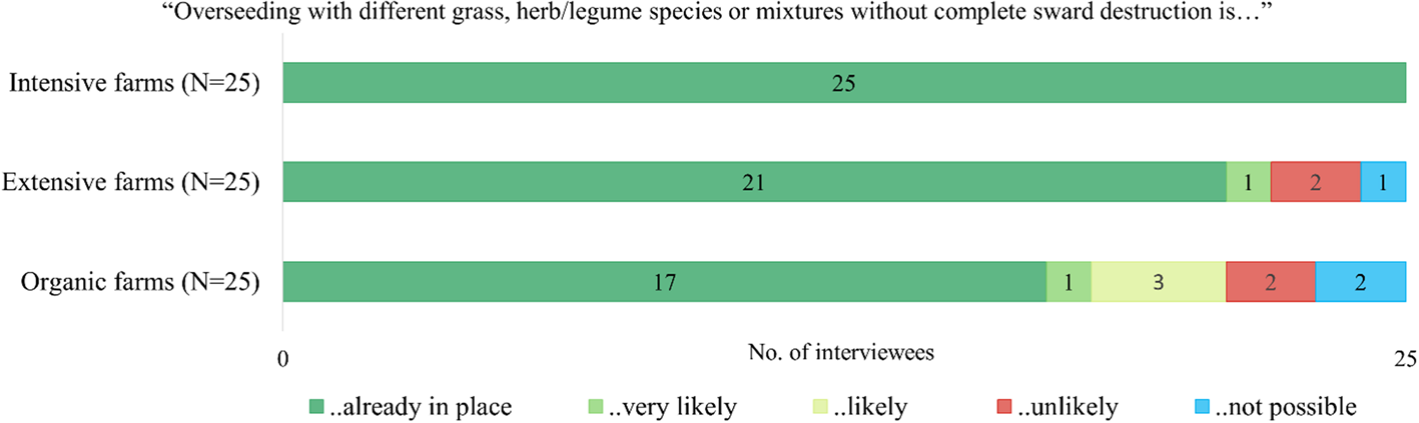
Results of farmer interviews: Uptake of overseeding in the Alpine region of Switzerland (Average stocking rate: Intensive farms = 1.7 LU/ha; Extensive farms = 0.7 LU/ha; Organic farms = 1.1 LU/ha)

The majority of the interviewees (63 of 75 interviewees) stated that overseeding was already in place. All interviewees with intensively managed PG said that they applied overseeding. Across the organic and extensive farm interviews, 21 interviewees with extensively managed and 17 with organic PG said that overseeding was already in place (Fig. 3). The Pearson *χ*^2^ test confirmed a significant difference between intensive and organic farms regarding current and future uptake of overseeding. Results of the Pearson *χ*2 test are reported in Appendix, Table 8.

Most farmers explained that they carry out overseeding on small areas of damaged grass, citing a number of reasons. One farmer stated that “*overseeding is carried out to fill in gaps in spring or where alpine sorrel was removed. Grass seeds are sown manually*” (CH-76). Another interviewee stated “*overseeding is required to reseed small damage*” (CH-59). A further interviewee mentioned “*massive sward damage caused by mice requires overseeding*”. The frequency of overseeding varied. For example, one farmer stated: “*I practice overseeding only occasionally and not every year” (CH-51).* Two others mentioned that *“overseeding is necessary every year because of sward damage caused by field mice*” (CH-55, CH-40). Another interviewee with intensively managed PG stated “*we carry out overseeding every year. We spend 500–1000 CHF every year on grass seeds to fill in the gaps and improve the sward*” (CH-32). Difficult seed germination and expensive seed drill machines were cited as limitations of overseeding in Alpine regions. One interviewee said that overseeding is not possible in Alpine regions “*it is difficult for the seeds to germinate. It would be much better if it is not necessary*” (CH-60).

The interviews also showed that the number of farmers who did not adopt any sward manipulating measure was small. All farms that adopted sward renewal, adopted also overseeding on sites where sward renewal was not possible or not necessary. In contrast, on farms where overseeding was not already in place, complete sward renewal was not practiced.

**Fig. 4:**
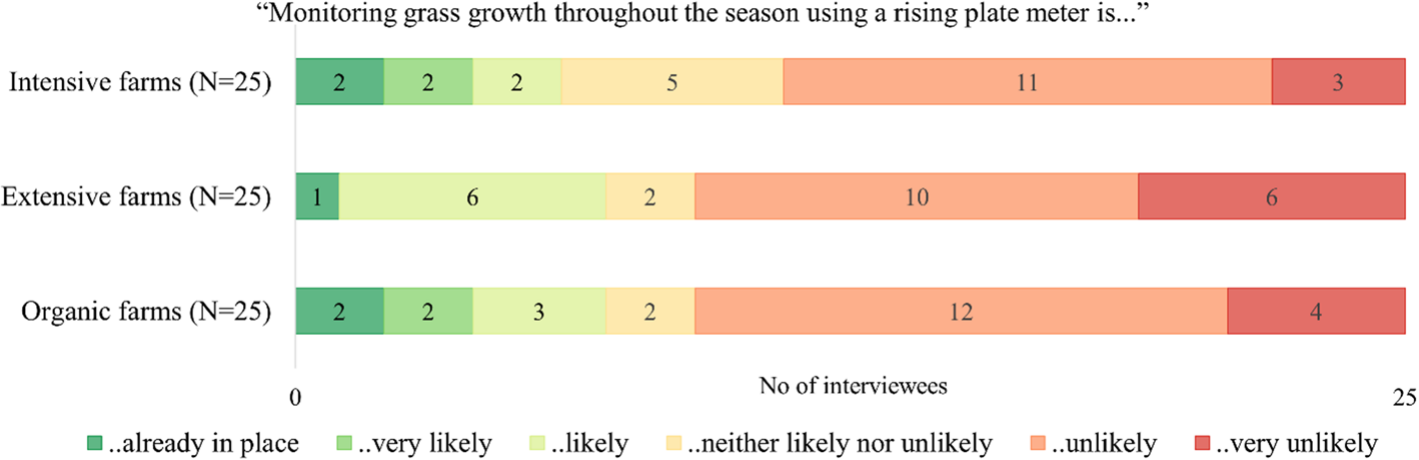
Results of farmer interviews: Use of rising plate meters in the alpine region of Switzerland (Average stocking rate: Intensive farms = 1.7 LU/ha; Extensive farms = 0.7 LU/ha; Organic farms = 1.1 LU/ha)

In the Swiss Alpine region, rising plate meters were not widely used (Fig. 4). Differences in the uptake between intensive, extensive and organic farms are small. The Pearson *χ*2 test did not show a significant difference in the use of rising plate meters between intensive, extensive and organic production farming systems. Results of the Pearson *χ*2 test are reported in Table 9 in appendix.

Limitations for the use of rising plate meters are the topography and associated grazing patterns in Alpine regions. One interviewee stated, for example, that “*rising plate meters are primarily used for continuous grazing but this is not possible [on my farm] because of the topography. We will continue with rotational grazing*” (CH-33). The performance of rising plate meters for PG, particularly on extensive farms, was questioned by one interviewee: “*rising plate meters work poorly on extensive grassland or permanent grassland, while they work well on temporary grassland with white clover and rye grass. This is my experience*” (CH-28). The majority of the interviewees stated that an uptake of rising plate meters was either “neither likely nor unlikely” or “unlikely” or “very unlikely”. Some interviewees indicated that they preferred to use their own judgement to assess grass growth, for example one farmer stated “*I use my eyes*” and another that “*I measure grass growth with my boots*” (CH-50).

### Feasibility of sward management options from an experts’ perspective

**Fig. 5:**
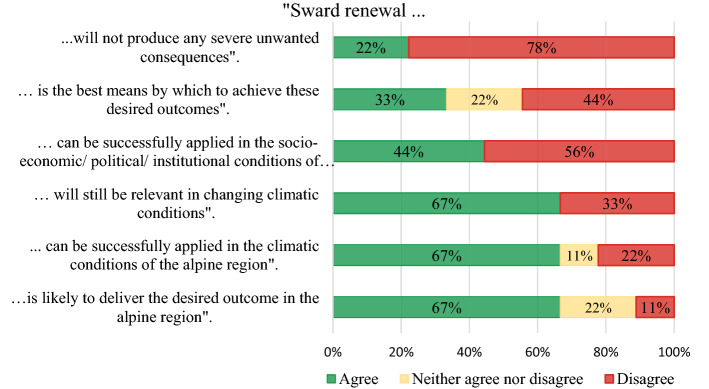
Results of the second round of the Delphi study: Applicability and feasibility of sward renewal on PG in Alpine regions of Switzerland (Total no. experts: *N* = 9)

In the Delphi study, consensus among experts regarding the feasibility of sward renewal in Alpine regions was measured by agreement or disagreement with six presented statements (see Fig. 5).Disagreement was highest for the statements “sward renewal will not produce any severe unwanted consequences” (78% of experts disagreed) and lowest regarding the statements “is likely to deliver the desired outcomes in the alpine region” (11% of experts disagreed). As shown in Fig. 5, most experts stated that sward renewal is a feasible management option under the climatic conditions of the Alpine region for PG and likely delivers the desired outcomes (to increase productivity and/or biodiversity). However, based on additional written information, experts also saw risks associated with sward renewal. For instance, one expert added “*Sward renewal would generally deliver the desired outcome. However, certain weeds can emerge and be particularly competitive at the early stages after renewal. Desired outcomes may not be achieved when seed mixtures are used that do not fit a site and when cultivation and management do not match*”. Another expert was much more pessimistic about the overall and medium- to long-term effect of sward renewal stating that “*existing species have developed in the grassland, because the conditions fit them. Alone, sward renewal is very unlikely to deliver any positive outcome in the medium term. This option makes no sense for Alpine regions*”. Another “disagreeing” expert added that sward renewal increases the risks of erosion and that no seeds adapted to the specific climatic conditions are available.

More than half of the experts (56%) stated that sward renewal cannot be successfully applied under the current political/institutional/socio-economic conditions in Switzerland. One expert added that destroying swards in mountain regions is not accepted by Swiss society. In addition, experts agreed that sward renewal is likely to produce severe unwanted consequences. One expert explained that: “*total reseeding should only be applied in very poor/unwanted sward compositions, since "bare soil" is always a risk for erosion and leaching, and it also sets free large amounts of carbon from the rotting old sward*”. Other examples stated included “*Erosion, nitrate leaching and carbon emission*”. Additional negative consequences caused by complete sward renewal were mentioned “*If not done well, it may cause erosion. Using imported seeds may also distort the regional gene pool*”. “*Complete sward renewal can induce nutrient losses to the environment, after turnover, which is not typical in grasslands*”, and “*Very high potential to negatively affect native species richness, soil erosion, especially in Alpine regions*”. These results show that most experts agreed that complete sward renewal was not a feasible or recommendable management option for PG in the Swiss Alpine region because of the high risk of negative effects on regulating, cultural and supporting ES.

**Fig. 6:**
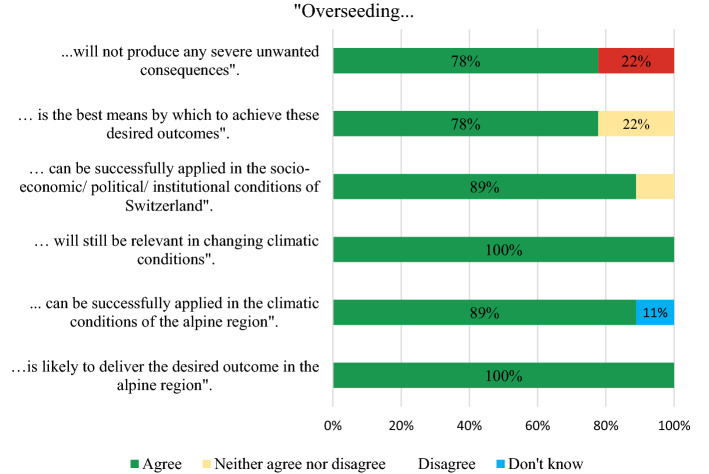
Results of the second round of the Delphi study: Applicability and feasibility of overseeding on PG in Alpine regions of Switzerland (total no. of experts: *N* = 9)

The experts reached consensus (defined as 80% and more of participants giving the same answer) on most of the statements in the survey regarding overseeding, indicating that overseeding represents a viable management option for PG in the alpine region (Fig. 6). One expert exemplified the ease of application and evidence of success as positive reasons for adopting overseeding: “*Establishing new species *via* overseeding is well tested and easy to apply while keeping a large part of the old sward to protect the soil and cap carbon emissions due to the soil disturbance*”. Conditions for success were also identified as being outside of the farmers control, for example another expert added that “*Not much can go wrong, except for weather conditions impairing establishment of new species (e.g. drought inhibiting germination of seeds)*”.

**Fig. 7:**
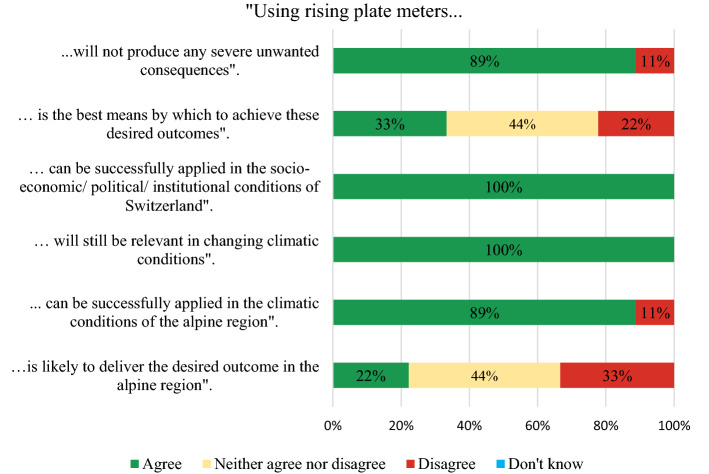
Results of the second round of the Delphi study: Applicability and feasibility of rising plate meters on PG in Alpine regions of Switzerland (total no. of experts: *N* = 9)

Experts reached consensus that rising plate meters will not produce any severe unwanted consequences (Fig. 7). One expert who disagreed added that this management practice “Could cost a lot of time and is not more precise than a visual assessment by the farmer”. Another expert stated that rising plate meters are not likely to deliver the desired outcome stated that “*In Alpine regions, grasslands are very diverse and it is very unlikely that the rising plate meter measurements will be properly calibrated for the very large range of situations*” (Fig. 7). In contrast, one expert mentioned that the use of rising plate meters “*has an additional benefit: the farmers to get to know the condition of their grassland and the sward diversity*”. One expert who disagreed that rising plate meters were the best means to achieve the desired outcomes mentioned “*I estimate the acceptance of the plate meter in practice as critical. The farmers having a "good eye" don't need one and the other ones probably will not use it*”.

### Expected effects of sward management options on ESs from an experts’ perspective

In the Delphi survey, experts were further asked for their expectations regarding the impact of sward renewal, overseeding and rising plate meters on different ES (see Table 2). Consensus was in-frequently achieved, which may reflect the different disciplinary backgrounds of the experts. For the purposes of this paper, we indicate when more than 50% of the experts stated that the management option was likely to have either a positive or negative or neither positive nor negative effect. A [+] in Table 2 is used to denote a positive effect, [−] a negative effect and [±] for agreement regarding neither a positive or negative effect.

**Table 2:** Results of the Delphi study: Effects that each management option is likely to have on delivery of ES provided by permanent grassland

	Sward renewal	Overseeding	Rising plate meters
*Regulating services*			
Biodiversity	[–]	[+]	[+/–]
Pollination	n.c	n.c	[+/–]
Carbon storage	[–]	n.c	[+/–]
Greenhouse gas emissions	n.c	[+/–]	[+/–]
Flood control	[–]	[+/–]	[+/–]
Water quality	[+/–]	[+/–]	[+/–]
Prevention of soil erosion	[–]	[+]	[+/–]
Prevention of soil compaction	n.c	[+/–]	[+/–]
Prevention of loss of organic soil matter	[–]	n.c	[+/–]
*Cultural services*			
Landscape aesthetics	n.c	[+/–]	[+/–]
Recreation	n.c	n.c	[+/–]
*Supporting services*			
Animal health and welfare	[+/–]	[+/–]	[+]
Provisioning services			
Grass production for livestock	[+]	[+]	[+]
Grass production for biomass	[+]	[+]	[+]

[+]: More than 50% of the experts stated that the management option is likely to have a positive effect

[+/–]: More than 50% of the experts stated that the management option is likely to have neither a positive nor a negative effect

[–]: More than 50% of the experts stated that the management option is likely to have a negative effect

n.c.: Experts achieved no consensus towards negative or positive effects

Overseeding was rated positively for biodiversity and prevention of soil erosion, while sward renewal was rated negatively for five out of nine ES (Table 2). In contrast, most experts stated that both overseeding and sward renewal have a positive effect on provisioning ES. The results of the Delphi study showed that measures for monitoring or predicting grass growth, such as rising plate meters, neither have a positive nor a negative effect on regulating ES. However, rising plate meters were rated positively in terms of supporting animal health and animal welfare, and provisioning services such as grass production for livestock and biomass. For sward renewal, a trade-off between regulating and provisioning services of PG was observed. In contrast, consensus was not achieved in relation to the cultural services of sward renewal. Overseeding is a management option with positive effects on both, regulating services and provisioning services. Experts included in this research indicated that overseeding is likely to have a positive effect on biodiversity and prevention of soil erosion and no negative effects on the other regulating ES.

## Discussion

The aim of this paper was to explore the feasibility of sward renewal, overseeding and rising plate meter use and their expected impacts on provisioning, regulating, supporting and cultural ES from PG in the Swiss Alpine region. This was conducted in order to better understand how farmers’ attitudes towards these three PG management practices may affect ES in the Swiss Alpine region. The delivery of ES depends on the natural production conditions and farmers’ management decisions such as livestock density or organic production. To consider these interrelations, interviews were carried out with non-organic farms whose stocking rate was above average of the participating farms (conventional intensive farms; *n* = 25), with non-organic farms with stocking rate below average (conventional extensive farms; *n* = 25) and organic producers (*n* = 25). Furthermore, expert knowledge about the feasibility of implementing the different management practices and their perceived effect on provisioning, cultural and regulating ES, helped to gain knowledge on their expected impacts on ES delivery in alpine regions. A broad range of experts from different disciplines were selected which were in the majority very familiar or fairly familiar with the three management practices. However, we did not know whether the experts had an extensive knowledge about the ES. Experts could only indicate when they perceived that they had no knowledge. In addition, the survey only covers agricultural management expertise. A Delphi survey of, NGOs, agricultural policy specialists and nature conservationists is required to further clarify the diversity of opinions on the different management options.

The results indicate that intensive grassland management linked to high stocking rates leads more frequently to severe sward damage that requires complete sward renewal in comparison with extensive or organic grassland management. These results confirm previous research, indicating that intensive grazing can actually lead to the degradation of both the soil and vegetation of grassland (Bilotta et al., 2007). It also confirms findings by Creighton et al. (2011) in Ireland that indicate grazing area renewal is affected by stocking rates. Farmer interviews in the Swiss alpine region show that ploughing or discing was used to destroy the sward, while herbicides were not applied. This might be because herbicide applications need special authorisation in Switzerland. The results confirm a study from Ireland where ploughing was also the most common method of sward renewal (Creighton et al., 2011). However, the uptake of sward renewal is limited in Alpine regions. On sites with unfavourable topographical and weather conditions, where ploughing or discing the sward is not possible, sward renewal is not feasible.

The views of farmers and experts regarding sward renewal were very similar, meaning that both the delivery of provisioning services and the potential harmful effects on other ES were identified as important. Even though 70% of the experts agreed that sward renewal can improve fodder production, the management practise is viewed as a threat to many other ES and thus was not recommended for the Swiss Alpine region.

Medium to long-term negative environmental effects were predicted by the expert group.^2^ These results confirm various recent studies which showed that sward renewal is increasing the risk of a release of soil organic C and N emissions to the environment and is enhancing sediment loss (Buchen et al., 2017; Kayser et al., 2018; Pulley et al., 2020; Reinsch et al., 2018).

Agricultural schools and farm advisors may increase farmers’ awareness about the negative effect of complete sward renewal on biodiversity, as biodiversity is an important feature of the grassland community. Furthermore, communication about new seed mixtures should emphasise that complete sward renewal should, in most cases, be avoided in favour of overseeding. If sward renewal is seen as the only possibility to repair a sward, the application of less disruptive approaches should be recommended.

Overseeding was an important management approach adopted by farmers in all farm groups. It was feasible for most of the interviewed farmers, and farmers did not raise any concerns about negative impacts on the environment. These results were in line with the expert assessments in the Delphi study. Experts judged that overseeding was successfully applicable under current climatic conditions and did not have any negative consequences. Furthermore, experts also expressed the view that overseeding does not have any negative trade-off between regulating and provisioning services. Indeed, effective overseeding with deep-rooting herb and legume species can improve the drought tolerance of some swards (e.g. Hofer et al., 2017). However, the purpose the overseeding needs to be considered. For example, if the purpose was to increase yield, then positive effects on regulating services might be limited.

Only a few farmers were indicated that they were using rising plate meters, or expressed an interest in using them in the future. Most farmers thought that rising plate meters were either not necessary or not feasible in alpine PG conditions. The farmers’ views regarding feasibility did not align with the experts’ assessments, who expressed the view that rising plate meters could be successfully applied under current climatic conditions in mountain regions. Only one expert agreed with the farmers that rising plate meters were not feasible because calibration in PG with diverse grassland species is too difficult. The importance of using equations calibrated for the botanical composition of the targeted type of swards is discussed by Hart et al. (2020).

## Conclusions

The results of farmers’ interviews regarding the uptake of sward renewal, overseeding and rising plate meters were combined with the results of a Delphi study. The latter involved experts evaluating the effects of these three management practices on ES delivery. The aim was to improve the understanding of whether specific PG management practices should be supported or not in the Swiss Alpine region. Although the use of complete sward renewal in the Swiss Alpine region may have predictable negative ecological consequences, around half of the interviewees had already adopted it on at least one of their grassland plots due to severe sward damage. Thus, agricultural schools and farm advisers could usefully provide information regarding how farmers can avoid sward damage that requires a complete sward renewal, and indeed under which circumstances complete sward renewal might entail lower risk for the delivery of some regulating ES. Overseeding represents a management practice that involves a lower level of risk from an environmental and economic point of view. Thus, the high adoption of overseeding which is currently practiced in the Swiss Alpine region (70–100% of the grassland farmers across the different sectors) could be maintained. Rising plate meters do not play a major role in grassland management in the Swiss Alpine region to date and will be unlikely to be used unless they are better adapted to diverse grassland botanical composition in the alpine region. In addition, the advantages of rising plate meters are not recognised by farmers in the Alpine region.

## Data Availability

Data will be made available on request.

## Appendix

See Tables 3, 4, 5, 6, 7, 8, 9.

**Table 3:** Relationship between altitude in m a.s.l. and indicative yield potential in Switzerland (Huguenin-Elie et al., 2017)

Management type and intensity	Indicative annual yield (t DM/ha)
*Meadows*	
Intensive	15.9–0.0058·altitude
Fairly intensive	12.1–0.0046·altitude
Low intensive	8.0–0.0032·altitude
Extensive	3.8–0.0015·altitude
*Pastures*	
Intensive	13.3–0.0046·altitude
Fairly intensive	10.1–0.0038·altitude
Low intensive	6.5–0.0026·altitude
Extensive	3.0–0.0012·altitude

Some indications about the management intensity classes can be found in Nemecek et al., (2011) (for the lower altitudes)

m.a.s.l. Meter above sea level

DM: dry matter

dt: dezitonne

**Table 4:** Description of the selected interviewees from the Swiss Alpine region

	Units	Organic farms	Intensive farms (non-organic)	Extensive farms (non-organic)	Total
Number of interviewees	No	25	25	25	75
*Regional distribution*					
Mountain zone 1	%	23%	48%	14%	29%
Mountain zone 2	%	25%	46%	48%	37%
Mountain zone 3	%	30%	6%	33%	25%
Mountain zone 4	%	22%	0%	5%	8%
*Farm characteristics*					
Stocking rate (Mean, SD)	LU/ha	1.1 (0.4)	1.7 (0.4)	0.7(0.2)	1.2 (0.5)
Total agricultural area (Mean, Stdv)	ha	29 (14)	32 (15)	48 (30)	37 (23)
Grassland (Mean, SD)	ha	26 (13)	25 (13)	32 (18)	28 (15)
*Farm type*					
Dairy cows	%	36%	80%	28%	48%
Suckler cows	%	20%	8%	32%	20%
Beef cattle	%	32%	12%	20%	21%
Horses/sheep and/or goats	%	12%	0%	20%	11%
*Socio-economic characteristics*					
Age (Mean, SD)	years	43 (12)	49 (9)	46 (12)	46 (11)
*Education*					
No vocational education and training or courses for part time farmers	%	0	0	8%	3%
Vocational education and training (VET): Federal VET diploma	%	28%	36%	52%	38%
Advanced federal diploma of professional education and training (PET)	%	56%	56%	20%	44%
Bachelor / Master or higher	%	16%	8%	20%	15%
*Language region*					
German	%	84%	92%	68%	81%
French	%	16%	8%	32%	19%

*LU* Livestock units, *PG* Permanent grassland, *SD*  standard deviation

**Table 5:** Selected ES from PG in the Delphi study. Source: Korevaar et al., (2019) (adapted)

Services	Output
1.1.1.Provisioning services	Fodder for livestock; fodder for biomass
2.2.2.Regulating services	Carbon storage, water quality, flood control, greenhouse gas emissions, prevention of soil erosion, prevention of soil compaction, prevention of loss of organic soil matter, pollination
3.3.3.Cultural services	Landscape aesthetics, recreation & tourism
4.4.4.Supporting services	Biodiversity, Animal health and welfare

**Table 6:** Number of experts and their familiarity with management practices (subjective judgement)

Management option	Total no. of experts	Familiarity with management options (No. of experts)		
		Very familiar^1)^	Fairly familiar^2)^	Not at all familiar^3)^
Sward renewal	9	5	3	1
Overseeding	9	5	4	0
Rising plate meters	9	4	3	2

^1)^I have a good knowledge of this option and/or I have personal/second-hand experience of using/ developing/ applying this management option

^2)^I had some knowledge/ awareness of this option but not in much detail

^3)^I hadn’t heard of this particular option before today

**Table 7:** Analysis of differences in the uptake of sward renewal between intensive/extensive and intensive/organic farms: Results of Pearson's Chi-squared (χ2) tests

Farm groups	N	Categories^a^	Pearson chi^2^	Pr
Intensive/Extensive	50	7^a)^	3.6908	0.718
Intensive/Extensive	50	2^b)^	2.0129	0.156
Intensive/Organic	50	7^a)^	3.5238	0.741
Intensive/Organic	50	2^b)^	2.0129	0.156

a: 7 categories: 1 = already in place; 2 = very likely [to adopt]; 3 = likely; 4 = neither likely nor unlikely; 5 = unlikely; 6 = very unlikely; 7 = not possible

b: 2 categories:: 1 = already in place; 1 = very likely [to adopt]; 1 = likely; 2 = neither likely nor unlikely; 2 = unlikely; 2 = very unlikely; 2 = not possible

**Table 8:** Analysis of differences in the uptake of overseeding between intensive/extensive and intensive/organic farms: Results of Pearson's Chi-squared (*χ*2) tests

Farm groups	N	Categories^a^	Pearson chi^2^	Pr
Intensive/Extensive	50	4^a)^	4.3478	0.226
Intensive/Extensive	50	2^b)^	2.0833	0.149
Intensive/Organic	50	5^c)^	9.5238	0.049
Intensive/Organic	50	2^d)^	6.8182	0.009

a: 4 categories: 1 = already in place; 2 = very likely [to adopt]; 3 = unlikely; 4 = not possible

b: 2 categories: 1 = already in place; 1 = very likely [to adopt]; 2 = unlikely; 2 = not possible

c: 5 categories: 1 = already in place; 2 = very likely [to adopt]; 3 = likely; 4 = unlikely; 5 = not possible

d: 2 categories: 1 = already in place; 1 = very likely [to adopt]; 1 = likely; 2 = unlikely; 2 = not possible

**Table 9:** Analysis of differences in the uptake of rising plate meters between intensive/extensive and intensive/organic farms: Results of Pearson's Chi-squared (*χ*2) tests

Farm groups	N	Categories^a^	Pearson chi^2^	Pr
Intensive/Extensive	50	6^a)^	6.6667	0.247
Intensive/Extensive	50	2^b)^	0.0891	0.765
Intensive/Organic	50	6^a)^	1.672	0.892
Intensive/Organic	50	2^b)^	0.3676	0.544

a: 6 categories: 1 = already in place; 2 = very likely [to adopt]; 3 = likely; 4 = neither likely nor unlikely; 5 = unlikely; 6 = very unlikely

b: 2 categories:: 1 = already in place; 1 = very likely [to adopt]; 1 = likely; 2 = neither likely nor unlikely; 2 = unlikely; 2 = very unlikely

## Footnotes

1 The «Arbeitsgemeinschaft zur Förderung des Futterbaues/Working Group for the Promotion of Forage Production» (AGFF) is an association of all farmers and institutions in Switzerland interested in forage production.

2 Please note, that consequences on ES might be different depending on which method is used: ploughing or herbicides. However, this was not captured by the Delphi survey.
